# A Review on the Molecular Dynamics of Enterotype *Bacteroides* 2 in Relation to Inflammatory Bowel Disease

**DOI:** 10.3390/ijms27114754

**Published:** 2026-05-25

**Authors:** Thuy Mi Nguyen, Anje A. te Velde

**Affiliations:** Tytgat Institute for Liver and Intestinal Research, Amsterdam University Medical Center (UMC), Amsterdam Gastroenterology Endocrinology Metabolism (AGEM), University of Amsterdam, Meibergdreef 69, 1105 BK Amsterdam, The Netherlands; tminguyen2002@gmail.com

**Keywords:** *Bacteroides* 2, bile acids, butyrate, diet, enterotypes, inflammatory bowel disease, pro-inflammatory cytokines

## Abstract

*Bacteroides* 2 (Bact2) is a dysbiotic enterotype often associated with susceptibility to developing diseases such as inflammatory bowel disease (IBD). Carriers of Bact2 are found to be less responsive to therapeutic treatments like vedolizumab. This enterotype is characterised by a large amount of *Bacteroides*, low diversity in bacteria, fewer butyrate-producing species, and generally a low abundance of microbes in the gut. However, it remains unclear whether this dysbiosis contributes to IBD pathology or if it is merely a result of inflammation in the gut. Due to its ability to influence treatment responses, it is crucial to understand the molecular mechanisms behind this enterotype, as well as the effect of diet on this dysbiosis. A high concentration of pro-inflammatory cytokine IL-1β was found in the faecal water of Bact2 patients, as well as an abundance of conjugated bile acids, whereas butyrate was found in decreased amounts. Through the consumption of a less industrialised diet, it could be possible to shift away from a dysbiotic enterotype like Bact2. This includes the consumption of whole-grain carbohydrates to increase the growth of butyrate-producers and maintaining a low-fat diet to decrease bile acid production.

## 1. Introduction

Inflammatory bowel disease (IBD) is a chronic inflammatory disease affecting the gastrointestinal tract, of which there are two subtypes: ulcerative colitis (UC) and Crohn’s disease (CD) [1,2]. Although the pathogenesis of IBD is complex, it is well established that interactions between genetic, environmental, microbial, and immune-mediated factors contribute to disease development. These factors mainly relate to the functioning of the intestinal barriers, immune response, and microbial dysbiosis [3]. The two subtypes differ primarily in their clinical manifestations. In UC, chronic inflammation occurs in the colon and rectum, whereas CD is characterised by patches of inflammation, known as skip lesions, that can occur anywhere in the gastrointestinal tract. Typically, diagnosis is established through a colonoscopy [4].

Besides inflammation in the intestine, an impaired epithelial barrier and mucosal layer is also associated with IBD [5]. The mucus plays an important role, as it is the first physical barrier that dietary compounds and bacteria come into contact with. It prevents the penetration of bacteria into tissues and also reduces inflammation in the gut. In patients with UC, the mucus layer is thinner compared with that in healthy individuals, causing the gut bacteria to be in closer contact with the epithelial barrier [6]. The epithelial barrier is another line of defence, containing enterocytes, goblet cells, and Paneth cells [5]. The specialised epithelial cells form both a physical and a chemical barrier by producing and secreting antimicrobial peptides. With dysfunctional intestinal barriers, it is more difficult to prevent inflammatory responses caused by the microbes present in the gut. There was decreased expression of these antimicrobial peptides found in patients with CD compared to healthy people [7].

The epithelium of the gut is bound together with tight junctions and adherens junctions, made up of proteins like zonula occludens 1 (ZO-1), claudin, and occludin, which interact with actin, present within the cell [8]. The tight junctions can be manipulated to allow certain soluble compounds to pass through the epithelium. This can be the result of stress or inflammation, as pro-inflammatory signals, like TNF-α, tryptase, or interleukins, can change the expression of claudins. Increased intestinal permeability allows bacterial antigens to enter the bloodstream. These antigens bind to toll-like receptors (TLRs) on antigen-presenting cells (APCs), thereby activating the innate immune system. These cells, such as macrophages and dendritic cells, originate from monocyte precursors located throughout the body, including the lamina propria [9].

For research on IBD in vivo, the dextran sodium sulphate (DSS)-induced colitis mouse model is one of the most common animal models used [10]. The oral administration of DSS does not have an influence on the synthesis of mucus but rather affects the existing mucus instead [11]. MUC2 mucin is part of the mucus structure and contains negative charges on both sialic acid and sulphate residues. Due to the formation of disulphide bonds, sulphated compounds such as DSS are easily soluble in this structure. This destabilises and rearranges the structure of the mucus. DSS is typically added to the drinking water of the mice, resulting in neutrophil migration, inflammation, erosion of the mucosa, and a shortened colon length [10]. Several environmental factors influence the susceptibility to DSS-induced colitis, such as the concentration and duration of the administered compound [12]. Additionally, the genetic factors of the mice, the microbiome, and the housing conditions play a role [10].

In order to understand the different microbial compositions in humans, they can be clustered into enterotypes to further analyse the microbiome [13]. In this manner, compositional differences can be related to health differences in individuals. Typically, these enterotypes are characterised and dominated by a key genus. The most common enterotypes are *Bacteroides* and *Prevotella*, which tend to be antagonistic. Specific bacterial species tend to be more suitable within one of these enterotypes. The former can be divided into two subtypes, known as *Bacteroides* 1 (Bact1) and *Bacteroides* 2 (Bact2). The Bact2 enterotype is seen as more dysbiotic, as it is associated with autoimmune and metabolic diseases like IBD, obesity, and diabetes type 2. The composition of this enterotype differs from that of Bact1. It is characterised by low gene diversity, an increased amount of the Enterobacteriaceae species, higher water content levels, and elevated saccharolytic fermentation activity [13]. Bact2 also shows lower levels of *Faecalibacterium*, butyrate producers, and overall beneficial commensal bacteria [14,15]. Lastly, the Bact2 enterotype is associated with a lower total microbial load than other enterotypes. It is also linked to systemic and gastrointestinal inflammation.

Although Bact2 is strongly associated with being a dysbiotic enterotype, the molecular mechanism behind this is unclear. Most importantly, it is still unknown whether the dysbiosis is the result of chronic inflammation or if whether plays a role in the pathology of IBD. The microbiome is a highly complex component, dependent on multiple environmental factors. The concept of enterotypes reduces the microbiome to a key species, and it is important to understand whether these are capable of influencing the pathology of IBD, as well as the treatment of the disease.

For this reason, the aim of this review is to establish the metabolic profile and microbial interactions of the Bact2 enterotype—in particular, we consider which of these factors could contribute to the progression of IBD and whether dietary factors influence this enterotype.

## 2. *Bacteroides* Species in Relation to IBD

One major component of the Bact2 enterotype is the presence of *Bacteroides* species. There are certain strains of *Bacteroides* that have an influence on the progression of IBD. *Bacteroides vulgatus*, for example, is able to synthesise proteases that influence the permeability of cells [16]. In Caco-2 epithelial cells, the transepithelial resistance (TEER) increased when these proteases were blocked with broad-spectrum protease inhibitors. Similarly, protease inhibition in *B. vulgatus*-monocolonised, IL10-deficient mice ameliorated the severity of colitis. These *Bacteroides*-originating proteases were also found to be overexpressed in UC patients. The abundance of these proteases is thought to originate from extracellular vesicles released in nutrient-rich environments. This disrupts the colonic epithelium, leading to an influx of immune cells such as neutrophils. This process further exacerbates existing colitis. However, *B. vulgatus* is not the only strain of *Bacteroides* with this effect. *Bacteroides fragilis* is known to produce a serine protease, known as Bfp1, which has the ability to cleave protease-activated receptor 2 (PAR_2_), thereby activating it [17]. The activation of this receptor increases intestinal barrier permeability and induces nociceptors, resulting in hyperexcitability and pain.

However, the abundance of the *Bacteroides* species does not automatically drive inflammation, as the Bact1 enterotype is not associated with dysbiosis. *Bacteroides* is a known beneficial commensal and does not only have pathogenic strains. The same species producing these proteases have been shown to exert protective effects against inflammation in the gut. Liu et al. (2022) showed that the oral administration of *B. vulgatus* in DSS-induced colitis mice resulted in the amelioration of colitis in the colon, with reduced mucin degradation, Throughout this review, several pro-inflammatory cytokines have been mentioned, as inflammatory cell infiltration, and epithelial damage [18]. The bacteria achieved this by manipulating the microbial composition in the gut, inducing the growth of anti-inflammatory and short-chain fatty acid (SCFA) producing-bacteria, like *Parabacteroides*, *Bacteroides*, *Anaerotignum*, and *Alistipes*. This suggests that *B. vulgatus* exerts beneficial effects within a microbial community, in contrast to its effects during monocolonisation in mice [16].

In the same study, *B. vulgatus* influenced the immune responses of LPS-stimulated macrophages in vitro by downregulating the expression of CD40, among others [18]. This gene plays a role in the activation of nuclear factor kappa-light-chain-enhancer of activated B cells (NF-κB), a key player in IBD inflammation due to its role in the induction of pro-inflammatory cytokines. Additionally, *B. vulgatus* increases SCFA levels through their innate ability to produce these beneficial compounds and to induce the growth of SCFA-producing microbes. Butyrate especially is a crucial metabolite that protects against inflammation by inhibiting NF-κB and maintaining the intestinal barrier.

Even though *B. vulgatus* shows both protective and inflammatory properties, *B. fragilis* has mostly been associated with inflammation in the context of IBD. The bacterium induces the differentiation of T-helper cell 17 (Th17) by inhibiting the release of exosomal microRNA (miRNA) derived from colon epithelial cells [19]. Usually, Th17 produces pro-inflammatory cytokines, like interleukin-17 (IL-17). miRNAs are able to influence molecular signalling by binding to the untranslated site of a target gene and regulating protein translational processes. miR-149-3p, in particular, can inhibit the differentiation of Th17. However, *B. fragilis* can downregulate the release of these miRNAs, adding to inflammation in the colon. Indeed, reduced levels of miR-149-3p in exosomes were found in IBD patients. The presence of *Bacteroides* does not only affect the pathogenesis of IBD but also obstructs the treatment of the disease. UC patients that were unresponsive when treated with a faecal microbiota transplant (FMT) showed a higher abundance of *Bacteroides fragilis* and *Bacteroides salyersiae* pre-treatment [20]. High taurine and hypotaurine concentrations were also found in non-responsive patients; these can be produced by genes found in these *Bacteroides* species. Taurine, in particular, can influence inflammation by regulating the NLRP6 inflammasome, crucial for the production of pro-inflammatory cytokines like IL-18.

Conversely, Xu et al. (2024) showed that the composition of the FMT donor also plays a role in the response to treatment [21]. When FMT was performed on DSS-induced colitis mice using donor faeces enriched with *Bacteroides thetaiotaomicron*, the success rate was higher, indicated by increased colon lengths and levels of tight junction proteins. *B. thetaiotaomicron* has been described several times to alleviate IBD symptoms. Li et al. (2021) found that administering this bacterium intragastrically in DSS-induced colitis mice reduced inflammation and attenuated colitis [22]. This was explained by its role in the regulation of the Th1/Th2 and Th17/regulatory T cell (Treg) ratios, which is usually disrupted in IBD patients. Enhanced levels of Th1 and Th17 cause the induction of pro-inflammatory cytokines like IL-17 and interferon (IFN)-γ and a decrease in anti-inflammatory cytokine IL-10. *B. thetaiotaomicron* balanced this ratio by suppressing IL-17 and IFN-γ while also increasing IL-10 in DSS-induced colitis mice. The sphingolipids in *B. thetaiotaomicron* also show a protective effect against inflammation [23]. These amino-alcohols act as signalling molecules to regulate the inflammatory system. Germ-free mice that were colonised with sphingolipid-deficient *B. thetaiotaomicron* showed the development of colitis due to the missing sphingolipid signals, which usually help the host to adapt to and tolerate the commensal bacteria. In patients with both UC and CD, the researchers also found reduced levels of these sphingolipids in faecal samples. Interestingly, *B. fragilis*-derived α-galactosylceramide, another sphingolipid, binds to antigen-presenting molecule CD1d to regulate the abundance of natural killer T cells. So, even this strain, previously described as having pro-inflammatory properties, can also aid in the training of the immune system, thereby preventing colitis.

**Figure 1:**
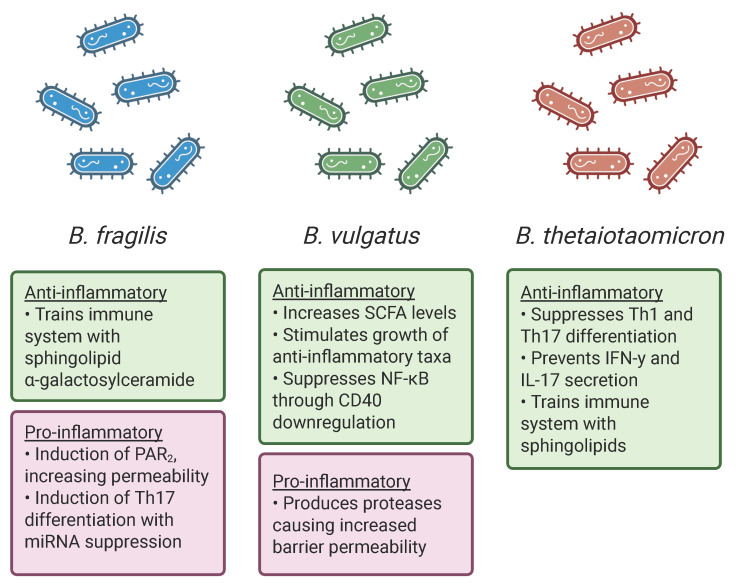
The roles of various *Bacteroides* strains in IBD. *B. fragilis* and *B. vulgatus* show anti-inflammatory properties (shown in green), as well as pro-inflammatory properties (shown in red). *B. thetaiotaomicron* mainly shows anti-inflammatory characteristics. Created in BioRender. Te velde, A. (2026) https://BioRender.com/q2gubpx.

It is clear that *Bacteroides* as a species is not solely pro-inflammatory and remains an important commensal in the gut. This is particularly evident in the absence of these species, causing susceptibility to inflammation. However, some species of *Bacteroides* are more strongly associated with pro-inflammatory activity, whereas others are more beneficial. *B. fragilis*, in particular, has several mechanisms that add to inflammation in the gut, whilst *B. thetaiotaomicron* shows mostly protective, anti-inflammatory properties (see Figure 1). However, the microbiome does not consist solely of one genus but of a complex community. For the Bact2 enterotype, lower alpha diversity and a generally lower microbial cell count are important characteristics in distinguishing it from the non-dysbiotic enterotype Bact1 [13]. Adding to this, Bact2 has also been associated with a lack of butyrate producers, such as *Faecalibacterium*, as well as a decrease in *Fuscobacterium* spp., *Streptococcus* spp., and *Veillonella* spp. [24]. These factors also largely explain why the Bact2 enterotype is so dysbiotic.

## 3. Low Microbial Diversity and Microbial Abundance in Relation to IBD

In addition to the presence of *Bacteroides* bacteria, low microbial diversity and an overall low microbial load are also characteristics of the Bact2 enterotype [13,24]. Through a meta-analysis across several studies, Rimmer et al. (2024) found a decrease in alpha diversity in the faeces of IBD patients when comparing them with healthy controls [25]. This pattern was observed even when comparing patients in remission with healthy individuals [26]. Similarly, a significant decrease in microbial load was observed in disease-affected individuals when using next-generation sequencing [27]. Notably, healthy relatives of UC patients showed an altered bacterial load compared to unrelated healthy controls. This suggests that genetic factors may contribute to a dysbiotic microbiome, increasing the susceptibility to developing UC.

The bacterial load can also serve as an indicator of inflammatory activity in the gut. Ventin-Holmberg et al. (2025) investigated the effects of infliximab (IFX) on the microbial composition in patients who responded and did not respond to treatment [28]. IFX is a monoclonal antibody targeting TNF-α, therefore inhibiting the cytokine and preventing further inflammation [29]. This therapeutic drug is able to restore the mucosal layer of the intestine in patients. Those that properly responded to IFX had a higher microbial load in their faecal samples after drug administration, as well as in their mucosal samples from the non-inflamed ileum. The opposite was seen in patients who were non-responsive to the drug, where no difference in microbial load was observed post-treatment. Importantly, mucosal samples were collected from different intestinal regions, including the non-inflamed ileum, non-inflamed colon, and inflamed colon. Within the responder group, the inflamed regions of the intestine already showed significantly lower microbial abundance compared to the non-inflamed sections. The effectiveness of therapeutic drugs is crucial in IBD, as treatment failure in acute cases may necessitate a colectomy, which carries substantial risks [30].

It is important to note that the above study suggests that the increase in microbial load is merely a result of the alleviation of inflammation following treatment. An inflammatory state in the gut can alter the mucosal layer, affecting the environment in which bacteria colonise the gut [31]. There is no indication that the microbial load can contribute to the severity of inflammation. Besides comparing longitudinal data within groups, it would be interesting to compare the bacterial loads in mucosal samples of non-responsive and responsive patients pre-treatment [28]. Such comparisons could reveal whether the baseline microbial load contributes to inherent differences in treatment responsiveness. Furthermore, when comparing the faecal samples between the two groups, the baseline level of bacterial abundance is similar. However, due to the local interaction of inflammation and IFX with the bacteria in the mucosa, faecal samples may not accurately reflect microbial dynamics during inflammation.

So, both low alpha diversity and a low microbial load have been associated with IBD and with the Bact2 enterotype. However, it is still not completely understood whether low diversity in microbes and a low bacterial count have a causal relationship with IBD or whether this is merely a result of intestinal inflammation. Even if these two factors influence the pathology of IBD, the mechanisms behind this are still unknown. Meanwhile, the vast genetic diversity of the microbiome suggests that greater microbial abundance and variance may lead to additional protective effects [32].

## 4. Metabolite Screening of Patients with the Bact2 Enterotype

A dysbiotic enterotype like Bact2 not only has a different microbial composition but also shows a different metabolic profile compared to other enterotypes. Poppe et al. (2024) screened UC patients with the Bact2 enterotype for metabolites and compared them to other patients [33]. Bact2 was present in 3.9% of healthy controls, compared with 31% of patients in remission (UC-R) and 68.6% of patients with active UC (UC-A). These carriers had a significantly higher IL-1β concentration in the faecal water compared with Bact1 and Ruminococcus enterotype individuals. The concentration of this pro-inflammatory cytokine negatively correlates with the concentrations of furan-containing compounds, which are known to have anti-inflammatory effects.

Bact2 carriers also generally showed higher amounts of taurine-conjugated bile acids, like taurochenodeoxycholic acid (TCA) and taurodeoxycholic acid (TDCA). Previously, these bile acids have been found to promote colitis in genetically susceptible mice [34]. It acts as an energy source for *Bilophila wadsworthia*, leading to the formation of H_2_S and the induction of a TH1 immune response. In Poppe et al. (2024), the concentration of bile acids correlated negatively with the faecal dry weight [33]. This suggests a fast passage rate, reducing metabolism and nutrient uptake, resulting in the malabsorption of bile acids. Besides this, they tested the faecal water regarding the functioning of epithelial cell barriers by measuring the change in TEER. Over time, TEER remained more stable in the faecal water of non-Bact2 carriers (84.1%) compared with Bact2 carriers (51.4%).

Short- and medium-chain fatty acids were also found in lower amounts compared to non-carriers, including butyrate. The faecal water of carriers showed lower gene expression of ACSM3 in HT-29 cells. This gene plays a role in preparing fatty acids for beta-oxidation by conjugating them to coenzyme A (CoA). Furthermore, ACAT2, involved in performing the last step of beta-oxidation, was overexpressed. Lastly, the intermediate metabolite maleic acid was also found in decreased amounts in Bact2 and IBD patients, whereas certain amino acids like tryptophan were increased.

To understand in greater depth how Bact2 affects the pathology of IBD, these altered metabolite profiles will be discussed in detail and related to intestinal inflammation. In particular, the pro-inflammatory cytokine IL-1β, the SCFA butyrate, and the role of conjugated bile acids are relevant to the mechanisms of IBD.

## 5. The Role of IL-1β in IBD

Throughout this review, several pro-inflammatory cytokines have been mentioned, as they play a relevant role in the pathogenesis of IBD. Through signalling cascades, they are able to influence inflammation; thus, it is logical that IL-1β has been found in higher amounts in Bact2 individuals [33]. This molecule is part of the interleukin-1 family, relevant to the innate immune system [35]. The release of these cytokines is triggered by pathogen- and damage-associated molecular patterns (PAMPs and DAMPs), as well as other pro-inflammatory signals like TNF-α. IL-1β, in particular, is an agonistic ligand, resulting in the differentiation of Th17 from CD4+ T-helper cells [35,36]. The expression of IL-1β is induced by TLRs, as well as by itself through a positive feedback loop. Interestingly, germ-free mice produced lower levels of the precursor pro-IL-1β, showing the importance of the microbiome in the activation of IL-1β and Th17 differentiation [36].

As mentioned previously, Th17 contributes to the progression of IBD due to its production of the cytokine IL-17 [37]. This signalling molecule can induce the secretion of inflammatory mediators and chemokines. Additionally, it promotes the release of granulocyte–macrophage colony-stimulating factor (GM-CSF), TNF-α, and proteases like matrix metalloproteinases (MMPs) in intestinal epithelial cells [37,38,39]. IL-17 is also able to induce the NF-κB and MAPK pathways. In turn, this results in the activation and migration of neutrophils, with the mucosal tissue as a target, leading to intestinal damage.

IL-1β is also able to increase the permeability of the intestinal barrier [40]. This permeability is induced through the NF-κB pathway. IL-1β promotes the degradation of IκB-α, resulting in NF-κB activation. Normally, upon the binding of IL-1β, the activation of NF-κB occurs through two pathways: the canonical and non-canonical pathways [41]. The canonical cascade uses mitogen-activated protein kinase kinase kinase-1 (MEKK-1) to activate inhibitory κB kinases (IκK), and the subsequent phosphorylation of the subunit IκK-β, leading to the degradation of inhibitory κB protein. This induces the production of NF-κB and causes its nuclear translocation, activating the target gene myosin light-chain kinase (MLCK). One of the proteins related to this gene, MLCK1, plays a role in the functioning of tight junctions. By phosphorylating the myosin II regulatory light chain (MLC), this protein is able to contract the actomyosin ring on the lateral side and retract the apical membrane, thereby being able to pull apart tight junction complexes [42]. This allows MLCK to render the intestinal barrier more permeable in an NF-κB-dependent manner. Besides the activation of the MLCK gene, the activation of NF-κB also results in the induction of other secondary cytokines like IL-6 and IL-8, causing further inflammation [43].

It is clear that IL-1β has an important role in intestinal inflammation and therefore the progression of IBD. One of the main regulators of this cytokine is the NLRP3 inflammasome, which is responsible for cleaving the precursor pro-IL-1β into its active form [35,44]. NLRP3 is primarily found in the macrophages of the intestinal mucosa and in dendritic cells. It is activated upon receiving two signals. The first signal is priming by pathogen recognition receptors (PRRs), which activates target genes including IL-1β. The second is activation by PAMPs and DAMPs via specialised membrane receptors, leading to the oligomerisation and formation of the NLRP3 complex. With this, the protease caspase-1 is generated, resulting in the eventual cleavage of pro-IL-1β into IL-1β.

**Figure 2:**
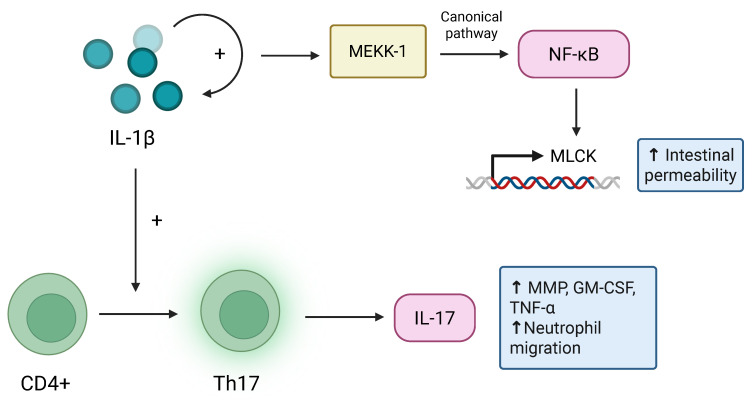
The involvement of IL-1β in IBD. Through the induction of MEKK-1, NF-κB is activated via the canonical pathway. This leads to the gene transcription of MLCK, resulting in an increase in the permeability of the intestinal barrier through pulling apart tight junction complexes. IL-1β also stimulates the differentiation of CD4+ T-helper cells into Th17 cells. These produce an abundance of IL-17, inducing the release of pro-inflammatory cytokines and neutrophil migration. Created in BioRender. Te velde, A. (2026) https://BioRender.com/19dgnoo.

To conclude, IL-1β is not only a product of existing inflammation within IBD pathology but also exacerbates the disease. It does so through a positive feedback loop involving the induction of pro-inflammatory cytokines and increased intestinal permeability (see Figure 2) [45].

As inflammatory cytokines play a major role in IBD, they have been the main target for therapeutic treatment. Interestingly, the Bact2 enterotype can influence the response to anti-inflammatory drugs. Caenepeel et al. (2024) investigated remission rates in IBD patients after the administration of anti-inflammatory treatments such as antitumor necrosis factor-α (a-TNF) or vedolizumab (VDZ) [15]. Here, 65.08% of Bact2 individuals experienced the alleviation of their symptoms after taking a-TNF. For VDZ, only 35.21% of the Bact2 patients entered into remission, showing that the drug was less effective for individuals with this dysbiotic enterotype. In addition to this, a-TNF therapy led to changes in the microbial composition as well, with 6 out of the 47 carriers shifting away from the Bact2 enterotype.

## 6. The Anti-Inflammatory Effects of Butyrate

A protective metabolite against the progression of IBD, which is also found to be decreased in Bact2 patients, is the SCFA butyrate [33]. In previous research, this dysbiotic enterotype has already been characterised by a lack of butyrate-producing microbes [15]. Butyrate is a common SCFA that is synthesised after carbohydrate fermentation by bacteria [46]. It assists in the strengthening of the intestinal barrier and activates the immune cells in the mucosal lining of the intestine, such as Tregs. This metabolite also inhibits the release of pro-inflammatory cytokines and chemokines that are derived from neutrophils, such as IL-6, TNF-α, and IFN-γ. Additionally, butyrate decreases the levels of proteins S100A8 and S100A9 and reduces the production of reactive oxygen species (ROS) and myeloperoxidase (MPO). The proteins S100A8 and S100A9 can form a heterodimer together, creating calprotectin, which has been used as a biomarker for the severity of inflammation and to predict mucosal healing in patients [47,48]. Compared to healthy individuals, calprotectin has been found in high concentrations in the faeces of IBD patients.

Butyrate also influences the migration of neutrophils, which plays an important role in the pathogenesis of IBD. Li et al. (2021) showed the effect of the inhibition of butyrate on the IL-8-dependent migration of neutrophils with the use of a transwell system and immunofluorescence [49]. Neutrophils in both healthy controls and IBD patients were taken and pre-incubated either with or without butyrate. In the transwell system, the lower chamber contained media with IL-8, usually the trigger for neutrophil migration. However, after incubation with butyrate, this migration did not occur. If there is an inflammatory site in the body, neutrophils migrate in its direction via signalling by cytokines or chemokines, like IL-8. Here, the immune cells degranulate, release toxins like ROS and MPO, and recruit more neutrophils [50]. The accumulation of these active immune cells causes further inflammation in IBD and can lead to cryptitis in acute severe UC. Additionally, this process of neutrophil migration prevents the restoration of the intestinal mucosa.

Upon activation, neutrophils are also able to form neutrophil extracellular traps (NETs) [49]. They serve to entrap bacteria and consist of a network of condensed DNA. However, in inflammatory diseases like IBD, these NETs are found to be accumulated in the mucosae of patients. These NETs also increased the damage to intestinal barrier integrity in DSS-induced colitis mice [51]. When treated with DNase I, the NETs dissolved, and the intestinal barrier damage was severely reduced in these animals. Furthermore, the levels of ZO-1 and occludin were shown to increase significantly after treatment with DNase I, meaning that the accumulation of NETs in DSS-induced colitis mice exacerbated the permeability of the gut barrier. Importantly, butyrate has the ability to inhibit the formation of NETs from neutrophils [49]. Both in vivo, with the use of DSS-induced colitis mice, and in vitro, by pre-incubating phorbol 12-myristate 13-acetate (PMA)-stimulated neutrophils with butyrate, reduced production of NETs was found.

Recently, butyrate has also been found to inhibit ferroptosis in intestinal epithelial cells (IECs) [52]. Ferroptosis is a form of cell death dependent on iron via mitochondrial atrophy, as well as lipid peroxidation and iron accumulation. This process has often been found to contribute to inflammatory diseases like UC by inducing damage to epithelial cells and endoplasmic reticulum (ER) stress-mediated IEC death. Butyrate is able to promote the expression of nuclear factor erythroid-related factor 2 (Nrf2), which is often disturbed in ferroptosis. Nrf2 can inhibit the production of ROS and iron accumulation and is essential to the inhibition of ferroptosis by butyrate. Butyrate administration in DSS-colitis mice resulted in an improvement in intestinal barrier integrity, and mitochondrial damage was reduced. In addition to this, Liu et al. (2025) showed inhibited ferroptosis in DSS-colitis mice through the increased phosphorylation of signal transducer and activator of transcription 3 (STAT3) [53]. This gene is phosphorylated by extracellular-regulated protein kinases 1/2 (ERK1/2), causing the inhibition of ROS production by mitochondria.

**Figure 3:**
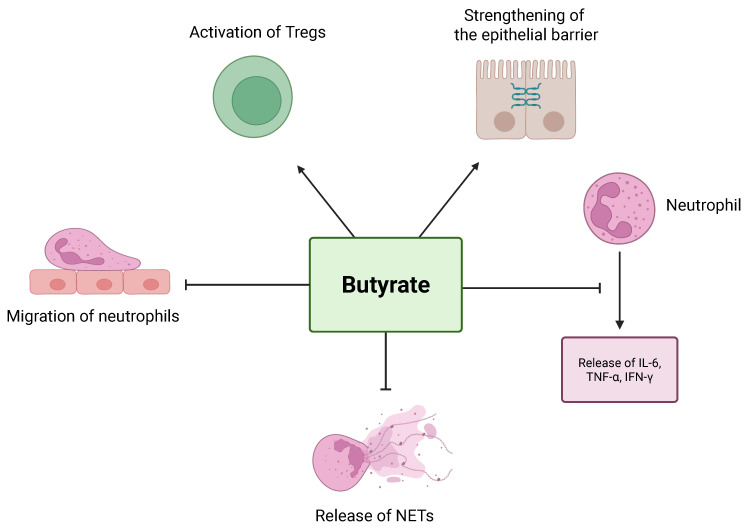
The role of butyrate in inflammation in IBD. Butyrate decreases inflammation in the gut by activating regulatory T cells and decreasing the release of pro-inflammatory cytokines like IL-6, TNF-α, IFN-γ, and NETs in neutrophils. This also results in the strengthening of the intestinal barrier. Created in BioRender. Te velde, A. (2026) https://BioRender.com/1mwo2ji.

Thus, it is clear that butyrate has a protective effect against inflammation by inhibiting the release of NETs and pro-inflammatory cytokines from neutrophils, as well as their migration to their target sites, thereby strengthening the intestinal barrier (see Figure 3). For this reason, the low levels of butyrate and butyrate-producing microbes in Bact2 individuals could potentially contribute to the aggravation of IBD.

## 7. Bile Acid Metabolism in Relation to IBD

Besides a lack of SCFAs, Bact2 carriers also seem to have dysregulated bile acid metabolism. Specifically, taurine-conjugated bile acids, like TCA and TDCA, were found to be in abundance in these individuals [33].

Bile acids are formed in the liver as a product of cholesterol metabolism [54,55,56]. These primary bile acids, like cholic acid (CA) and chenodeoxycholic acid (CDCA), are stored in the gallbladder and are sent to the intestine. Prior to storage, bile acids can be conjugated with taurine or glycine, leading to the formation of bile by mixing with phospholipids and cholesterol. Bile is released into the intestine, where its main function is to form micelles with dietary fats to render the fats soluble and easy to digest. After the fats are absorbed, the bile acids are recycled after they are taken up in the intestine, where they are sent back to the liver. The bile acids that are not absorbed are transformed in the gut by microbes into secondary bile acids, with deconjugation being one of the first steps [56,57]. An important enzyme involved in this process is bile salt hydrolase (BSH), found in species like *Bacteroides*, *Clostridium*, *Bifidobacterium*, and *Lactobacillus*. Secondary bile acids are able to present themselves in different isomeric forms due to modifications, which may exert an influence on inflammatory, immune, and endocrine homeostasis. Both primary and secondary bile acids are able to activate the farnesoid X receptor (FXR) pathway in their conjugated and unconjugated forms. The activation of this nuclear receptor mainly assists in the metabolism of lipids and glucose, but also in the inflammatory and apoptotic pathways [57]. Conjugated bile acids are hydrophilic and require the usage of the ileal bile acid transporter (IBAT) to cross the membrane [58]. In the absence of this transporter, the conjugated bile acids are unable to activate the receptor. This is not the case for unconjugated bile acids. Due to their high p*K_a_*, these compounds can undergo non-ionic passive diffusion across membranes [59]. Additionally, they have been found to have a stronger affinity for FXR compared to conjugated bile acids [60].

When FXR is activated, it binds with Retinoid X Receptor (RXR) to its response elements on target genes, like small heterodimer partner (SHP), intestinal bile acid-binding protein (IBABP), and fibroblast growth factor 15 (FGF15) in mice and fibroblast growth factor 19 (FGF19) in humans [61]. Many studies have reported that FXR activation has protective effects against inflammation. An example of this is the activation of the SHP gene, which inhibits the formation of the NLRP3 inflammasome, causing a decrease in pro-inflammatory cytokines. The production of IL-6 is also inhibited in macrophages upon activation of the FXR receptor [62]. Moreover, CDCA-dependent activation of FXR leads to binding to the promoter region of the monocyte chemoattractant protein-1 (MCP-1) gene, causing its inhibition. This gene is primarily produced by macrophages, and it induces the infiltration and migration of macrophages. Besides the reduction of these inflammatory pathways and cytokines, the production of tight junctions has also been described to increase upon activation [63,64,65]. While FXR activation is able to influence the production of pro-inflammatory cytokines, the opposite is also true. Signalling molecules are able to inhibit the FXR pathway, leading to the exacerbation of existing inflammation [61].

Poppe et al. (2024) reported a high abundance of conjugated bile acids in Bact2 patients, observing the decreased activation of FXR due to malabsorption [33]. Interestingly, Yang et al. (2025) claimed the opposite, where a high abundance of unconjugated bile acids was found in DSS-induced colitis mice because of an increase in BSH-producing bacteria [63]. Their hypothesis was that, due to the lack of conjugated bile acids, the FXR receptor is unable to be activated. However, previous research has established that unconjugated bile acids also contribute to FXR activation [57,60], so it is interesting that an abundance seemingly decreases the functioning of the receptor.

An explanation for these results in Yang et al. (2025) could be the negative feedback loop provided by the FXR pathway through the inhibition of IBAT and endogenous bile acid synthesis by the liver [63]. When administering the bile acid obeticholic acid (OCA) in humans for 17 days, Friedman et al. (2018) showed that the FXR agonist led to an overall decrease in bile acid production [64]. This inhibition was caused by the downstream effector of the FXR pathway, SHP, preventing the rate-limiting step of bile acid synthesis from cholesterol, performed by the cytochrome P450 enzyme [66]. Thus, bile acids inhibit their own production through a feedback loop. Interestingly, Xu et al. (2021) supplemented mice with the bile acid deoxycholic acid (DCA) for six months, resulting in an increase in unconjugated bile acids, even though BSH expression decreased [67]. The duration of the trial, as well as different model organisms, could explain the differences between Friedman et al. (2018) and Xu et al. (2021) [64,67]. Another way in which the FXR pathway can regulate bile acid synthesis is by inhibiting IBAT [66,67]. Thus, chronic exposure to DCA could have resulted in the inhibition of IBAT, therefore causing a decrease in FXR activation. This halts the negative feedback loop in endogenous bile acid production, increasing its abundance.

**Figure 4:**
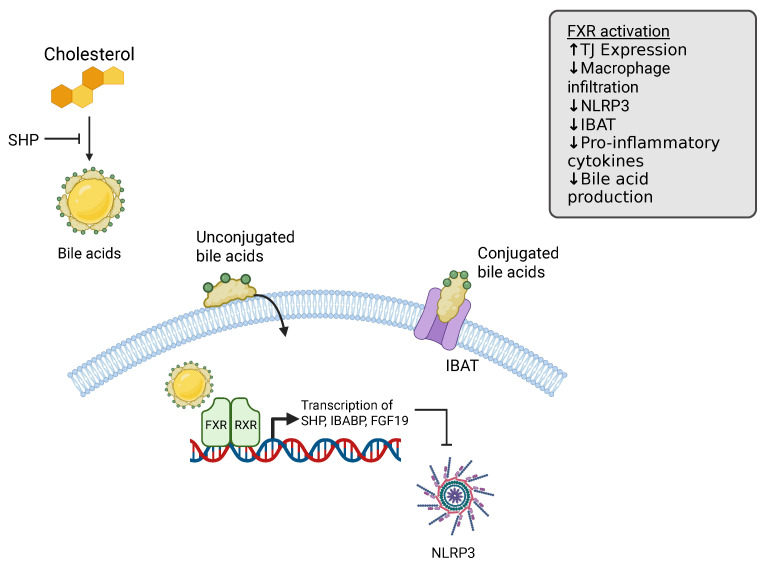
The FXR activation pathway. Conjugated and unconjugated bile acids are able to activate the FXR receptor, with the first utilising IBAT, whereas the latter can pass through the membrane. Upon activation, FXR binds together with RXR to induce the transcription of SHP, IBABP, and FGF19. The presence of SHP inhibits both the formation of the NLRP3 complex and the production of bile acids. The activation of FXR also leads to the inhibition of IBAT, the inhibition of macrophage infiltration, and the expression of tight junctions (TJs). Created in BioRender. Te velde, A. (2026) https://BioRender.com/dc6wmxt.

This suggests that dysbiosis in bile acid synthesis skews the homeostasis of the FXR pathway. Both chronic overactivation and inhibition due to malabsorption lead to inflammatory conditions in the gut (see Figure 4). In the case of Bact2 individuals, this suggests that the observed malabsorption of bile acids could play a role in the pathogenesis of IBD [33].

A recurring finding is a reduction in secondary bile acids in the faeces of IBD patients, regardless of the conjugated form [56,68]. Secondary bile acids can have anti-inflammatory effects by preventing the secretion of pro-inflammatory cytokines TNF-α, IL-1β, and IL-6 in macrophages. In the intestine, bile acids can be sulphated through the epithelium, whereas microbes in the gut can cause desulphation. Duboc et al. (2013) found that the sulfatase enzyme in the faeces was lower in activity in IBD patients [68]. When secondary bile acids are sulphated, it is at the cost of their anti-inflammatory effects. This means that, due to a higher ratio of sulphated/unsulphated secondary bile acids, there is a reduction in their anti-inflammatory properties in IBD patients.

Contrary to examining the conjugated/unconjugated bile acid ratio, Lu et al. (2025) looked further into the malabsorption of bile acids [69]. They claim that IBD patients experience an overflux of bile acids in general, as reabsorption is disturbed due to the inflammation of the ileum. Again, an abundance of sulphated bile acids is mentioned, which exacerbates bile acid malabsorption (BAM). Interestingly, FMT, often used as therapy for IBD, was more successful in patients that experienced BAM compared to those who did not. This suggests that a dysbiotic microbiome was relevant to the impaired bile acid metabolism in the BAM group and therefore the IBD pathology. In contrast, the non-BAM group showed colitis despite FMT, indicating another factor besides the microbiome that is at play.

It is important to note that, regarding the studies examining human subjects (see Table 1), factors such as diet and antibiotic use were not accounted for [33,64,68,69]. As these factors can influence the microbiome, and consequently bile acid metabolism, these variables must be considered.

## 8. The Roles of Saccharolytic and Proteolytic Fermentation in IBD

In a previous paper, another characteristic of Bact2 that was mentioned was an increase in saccharolytic activity [13]. The Bacteroidetes phylum in general can degrade a wide range of complex carbohydrates that are indigestible by humans [70]. Other papers, however, have reported the general *Bacteroides* enterotype to have increased proteolytic activity, in addition to saccharolytic activity [70,71].

Fermentation in bacteria can occur through the use of carbohydrates, proteins, or lipids as an energy source [72]. In the human colon, saccharolytic fermentation is preferred by bacteria over proteolytic fermentation. It seems that proteolytic fermentation occurs mainly in the distal colon when carbohydrate availability is depleted. When moving distally in the colon, the pH becomes more neutral, contributing to the induction of the proteolytic fermentation of bacteria. Supporting this, phenolic compounds derived from the degradation of aromatic amino acids were found in a four-fold higher concentration in the distal colon compared to the proximal colon [73].

Interestingly, concerning IBD and the fermentation type, De Cruz et al. (2015) reported finding mainly bacteria that preferred proteolytic fermentation and lactic acid production in patients with CD [74]. These microbes have been associated with the accumulation of end-products that are known to be toxic to colonocytes, like NH_3_, CH_4_, H_2_, CO_2_, and H_2_S, possibly contributing to colonic inflammation [29,75]. H_2_S, in particular, is an important driver of IBD. This molecule is able to inhibit beta-oxidation, impairing epithelial metabolism and raising oxygen levels in the lumen [76]. The result would be an unfavourable environment for anaerobic bacteria that includes many butyrate producers. A shortage of these microbes consequently leads to a decrease in butyrate. The degradation of proteins has also previously been linked to the growth of *Bacteroides* species, as well as the growth of *Clostridium* spp. [75]. In contrast to patients with active disease, those who were in remission with IBD showed higher levels of saccharolytic species [74]. These bacteria are able to produce SCFAs like butyrate from resistant starch and fibre, again protecting the gut from inflammation.

Overall, proteolytic fermentation appears to be linked to inflammation, whereas saccharolytic fermentation shows protective effects. This does not align with the fact that the Bact2 enterotype—the supposedly dysbiotic enterotype—has been described as being more saccharolytic by Bresser et al. (2022) [13]. However, aside from the above review, there is no indication that the Bact2 enterotype displays exclusively increased saccharolytic fermentation. Interestingly, the above authors reference a study conducted by Vieira-Silva et al. (2019), who previously associated the *Bacteroides* enterotype with an increase in both proteolytic and saccharolytic fermentation [14]. Moreover, Rist et al. (2013) stated that proteolytic fermentation drives the growth of *Bacteroides*, whereas it has the opposite effect for butyrate-producing species [75]. Because of this, diet also has an important role in IBD and the behaviour of Bact2. Proteolytic activity is directly linked to the availability of carbohydrates, so an individual with this dysbiotic enterotype could exacerbate the inflammation by consuming a carbohydrate-poor diet.

## 9. How Diet Influences the Functioning of Microbiota and Their Metabolism

Food is one of the most crucial environmental factors influencing the composition of the gut microbiota. However, is it possible for the diet to influence the microbiome in such a way that it can shift away from the Bact2 enterotype to a more favourable one?

Bresser et al. (2022) described that the most prevalent enterotype differs based on geographical location [13]. Areas where the diet typically consists of non-industrialised, fibre-rich foods, like rural populations in Africa, South America, and South-East Asia, had a high prevalence of the Prevotella enterotype. In contrast, the Bact1 and Bact2 enterotypes have been found in North America and urbanised areas, showing a more dysbiotic microbiome.

Alili et al. (2022) conducted a study investigating how diet can influence the Bact2 enterotype, in which obese individuals followed a diet program by the RNPC program (Rééducation Nutritionnelle et Psycho-Comportementale) [77]. They found that Bact2 individuals had low microbial diversity and were more likely to have a higher BMI and unhealthy metabolic biomarkers such as elevated triglycerides and liver transaminases. Furthermore, only individuals with low microbial diversity showed a shift in enterotype, including 10% of baseline Bact2 individuals. The dietary program consisted of three phases: (1) a carbohydrate-deficient, calorie-restricted diet with an abundance of proteins, including commercial protein-enriched food products; (2) protein-enriched foods were reduced, and the amounts of whole foods were increased, for low-glycaemic-index carbohydrate intake; (3) lastly, the weight maintenance phase, where the energy intake was the same as the post-weight-loss energy requirement.

Thus, the diet is indeed able to influence the microbial enterotype. It is important to note that the above study focused only on patients with obesity aiming at weight loss, rather than on IBD. Interestingly, as discussed previously, a lack of carbohydrates in the diet mainly leads to proteolytic fermentation in the gut, which has been linked to growth in *Bacteroides* species and a decrease in butyrate-producing species [72,75]. This is in contrast to Alili et al. (2022), where a high-protein and carbohydrate-deficient diet reduced the prevalence of the Bact2 enterotype [77]. Other factors could have been at play—for example, the fact that the diet was also calorie-restricted and that it shifted from processed commercial foods to whole foods. Furthermore, a high abundance of *Bacteroides* combined with low microbial diversity has been linked to the consistent consumption of meat, mostly found in areas like Europe and North America [72]. In contrast, plant-based diets, found in South American, African, and Asian populations, led to a decrease in this species. However, it is the abundance of dietary fibre that leads to an increase in butyrate-producing bacterial species, rather than the absence of meat.

Supporting this, Faits et al. (2020) compared participants undergoing three types of carbohydrate diets: simple, refined, and unrefined carbohydrates [78]. The simple carbohydrate diet consisted of a high abundance of foods with sucrose or high-fructose corn syrup. The refined carbohydrate diet consisted of refined grains like white rice, white bread, and white pasta. Lastly, the unrefined carbohydrate diet contained foods derived from whole grains. They found that a butyrate-producing species, *Roseburia*, was prevalent among participants consuming the unrefined diet compared to the simple carbohydrate diet. Alpha and beta diversity did not change significantly when looking at all patients overall; however, there were two patients that experienced a shift in microbial diversity when changing diets. This highlights how the effects of treatments on individuals differ regarding the microbiome. The main difference between whole-grain carbohydrates and refined grains is that, in the former, the bran and germ fractions of the grains are retained, whereas, for the latter, these parts are discarded during processing [79]. In the bran and germ, all nutrients of the grain are present, such as vitamins, minerals, phytochemicals, and dietary fibre. This fibre is indigestible in humans, which microbes take advantage of, causing a surge in growth as they use these carbohydrates as substrates.

Besides carbohydrates, it seems that dairy also shows protective effects against inflammation in the gut. Zhai et al. (2019) researched the effects of low-fat yoghurt on intestinal barrier permeability, induced by IL-1β [80]. Using a monolayer of Caco-2 cells, 25% dilutions of non-fat and low-fat yoghurt were applied after IL-1β stimulation. They found that both non-fat and low-fat yoghurt inhibited the reduction in TEER levels caused by IL-1β. Furthermore, low-fat yoghurt specifically reduced paracellular translocation, as well as the production of cytokine IL-8. The protective effects of yoghurt are thought to be related to its abundance of SCFAs. This could also explain why low-fat yoghurt showed more beneficial effects compared to non-fat yoghurt.

In line with this, a recent study by Yang et al. (2025) showed the anti-inflammatory effects of donkey milk on DSS-induced colitis mice [81]. They found that, after the intake of the milk, inflammatory symptoms, like colon damage and intestinal permeability, decreased. The downregulation of genes like ZO-1, occludin, and MUC2 after DSS administration was reversed when treated with donkey milk. Normally, DSS-induced colitis leads to an increase in Bacteroidota and reduced Firmicutes levels. Donkey milk administration counteracted this, increasing the Firmicutes/Bacteroidota ratio. Many species belonging to the Firmicutes phylum are SCFA producers, which could explain the beneficial effects of the treatment. Besides this, several metabolic pathways related to cofactors, vitamins, and amino acids were found to be upregulated due to the intervention. Furthermore, dairy consumption was able to reduce the levels of IL-1β, IL-18, and TNF-α, meaning that it dampens the NF-κB signalling pathway. It is not clear, however, whether donkey milk specifically has nutrients that ameliorate inflammation or if this is the case for dairy in general. Interestingly, besides being anti-inflammatory, the whey protein from donkey milk has been shown to have antimicrobial, antiproliferative, and antioxidant effects as well [82]. Importantly, the production yield of milk in donkeys is significantly lower than in other animals. Moreover, different breeds of donkeys show high variety in their milk compositions. Zhai et al. (2019) concluded that low-fat yoghurt was more favourable against intestinal inflammation compared to non-fat yoghurt [80]. However, the latter still showed some beneficial effects, suggesting that dairy in general seems to help to alleviate inflammation in IBD. As IL-1β levels are elevated in Bact2 patients, it is plausible that increasing dairy intake, such as through low-fat yoghurt, may be advantageous for these individuals.

Lastly, diets containing high levels of fat exacerbate the pathology of IBD. Xu et al. (2021) mimicked a Western diet by administering DCA bile acids in the diets of mice, which resulted in IBD symptoms [67]. This was supported by Islam et al. (2011), where a high-fat diet led to an increase in bile acid secretion to support the facilitation of lipid metabolism in rats (see Table 2) [83]. Here, different concentrations of CA, 1.25 mmol/kg (M-CA) and 5 mmol/kg (H-CA), were administered in rats to imitate a high-fat diet. They found a decrease in overall bacterial cell density and lower alpha diversity, and *Enterobacteriaceae* were more tolerant to the high abundance of bile acids. Bile acids are known to have antimicrobial properties, with the primary mechanism being the disturbance of bacterial membranes. This is likely the reason for the change in diversity and density of the microbiota. Conjugated bile acids were already found to be increased in Bact2 patients as well [33], suggesting that the consumption of a high-fat diet could aggravate their inflammatory responses.

## 10. The Relevance of Enterotypes in Clinical Applications

As discussed throughout this review, the Bact2 enterotype shows a dysbiotic microbial composition, suggesting an increased susceptibility to developing IBD. However, could this be relevant in the broader context of IBD research or potential therapeutic treatment? Moreover, how do the other enterotypes align with this?

**Figure 5:**
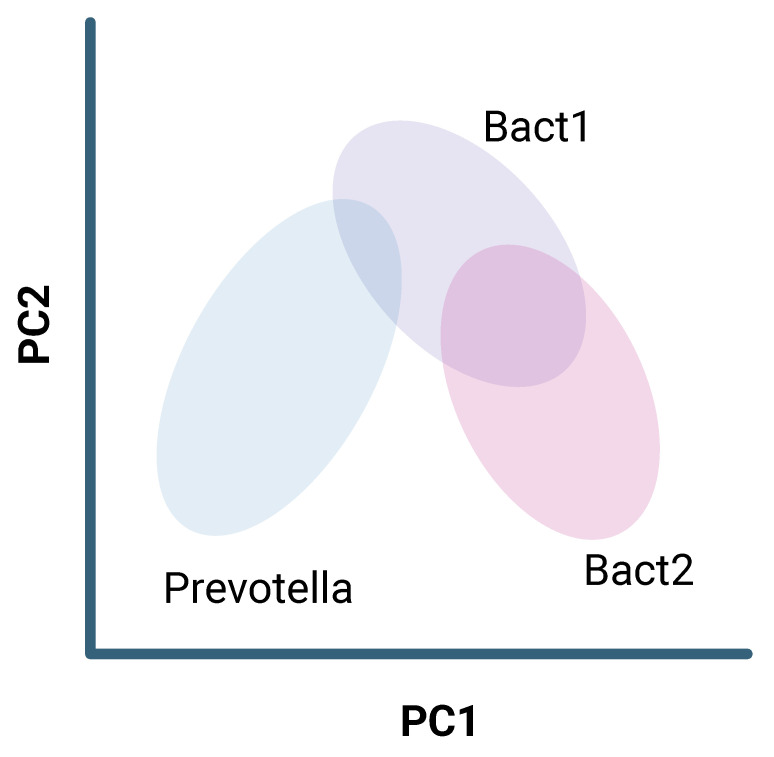
A model of a principal component analysis (PCA) visualising the overlap of enterotype clusters. This analysis shows the similarity between the microbial compositions of grouped individuals. In clustering, it is ideal for individuals to be clearly separated. In actuality, these enterotypes will overlap in several ways.Created in BioRender. Te velde, A. (2026) https://BioRender.com/6s3vuqa.

The first description of enterotypes separated the microbiome into two clusters, *Prevotella* and *Bacteroides*, or three clusters, where *Ruminococcus* was added [84,85]. Since then, there have been several variations of the enterotype clustering framework. An example is the creation of subtypes for *Bacteroides* (Bact1 and Bact2). There are papers that distinguish up to seven different enterotypes, describing the subtypes of *Bacteroides* (B1-1, B1-2, B2), *Ruminococcus* (R1, R2), and *Prevotella* (P1, P2) [24]. Due to the large number of ways in which enterotypes can be grouped together, there has been criticism of the clustering approach to the microbiome [84]. Grouping profiles together in different manners can highlight other aspects of health. For this reason, a gradient model has also been proposed as an alternative. Supporting this is the grouping of enterotypes and the fact that they are not entirely separated from each other in reality [86]. In theory, when performing a principal component analysis (PCA), it would be ideal if these enterotypes were clearly separated from each other so as to emphasise the differences in their composition. However, in actuality, there will be some overlap between the different enterotypes, resulting in a continuous gradient (see Figure 5). This distinction becomes more complex for subtypes of an enterotype, like Bact1 and Bact2.

Apart from environmental factors, age can also be an important factor that drives enterotype clustering. Different life stages show prevalences of various enterotypes, such as young school-aged children mainly having the Bifidobacterium enterotype in place of the Ruminococcus enterotype [87]. The microbiome in the elderly is also found to be extremely dynamic due to changes in lifestyle and diet and an increase in antibiotic use [86]. Compared to young adults, the elderly were found to have a distinct core microbiome, with a higher prevalence of the *Bacteroides* enterotype, as well as a higher abundance of Clostridium clusters [88].

## 11. Discussion

One of the characteristics of the Bact2 enterotype reviewed in Bresser et al. (2022) is its increased saccharolytic fermentation [13]. However, upon closer examination of the cited evidence, the referenced study by Vieira-Silva et al. (2020) [89] does not appear to address *Bacteroides*-related fermentation. Another study by Vieira-Silva et al. (2019) examined the fermentation processes of several enterotypes in depth [14]. Still, an increase in both proteolytic and saccharolytic fermentation was associated with the general *Bacteroides* enterotype. Therefore, presenting the Bact2 enterotype as being primarily characterised by carbohydrate fermentation may be an oversimplification. Inconsistencies were observed not only in the fermentation profile of Bact2 but also in studies on bile acid metabolism. First, Poppe et al. (2024) and Yang et al. (2025) showed altered results when observing the bile acid abundance in IBD pathology [33,63]. The former stated an increased concentration of conjugated bile acids in Bact2 patients, and the latter claimed a higher abundance of deconjugated bile acids in DSS-induced colitis mice, due to BSH-producing bacteria like *Bacteroides*. These findings suggest that both overactivation and insufficient activation of the FXR receptor may contribute to intestinal inflammation. However, the differences in investigating the metabolic profiles of Bact2 in humans compared to colitis-induced mice could also have played a role in these contradictory results. The same incongruity is observed in the papers by Friedman et al. (2018) and Xu et al. (2021), where they administered bile acids to human individuals and mice, respectively, mimicking the Western diet [64,67]. The first showed a decrease in bile acids whereas the latter showed an increase in bile acids after treatment. Another variable influencing these contradictory results is the duration of the intervention, where the experimental period only lasted 17 days for Friedman et al. (2018), whilst it spanned a total of 6 months for Xu et al. (2021) [64,67].

It is clear that there are a number of factors modulating the microbiome, as diet influences the behaviour of bacterial metabolism. Host dysmetabolism can also affect the microbiota, as discussed regarding the bile acid metabolism pathway. However, the opposite is also true. An example of this is the desulphation of bile acids, performed by the gut bacteria. In IBD, sulfatase activity is decreased, resulting in an abundance of sulphated secondary bile acids. The result of this is the loss of their anti-inflammatory properties. Additionally, the decreased desulphation could also be influenced by the low microbial load, as well as the low diversity of bacteria, as in the Bact2 enterotype. Due to the large number of factors influencing microbiome maintenance, *Bacteroides* as a genus does not exhibit solely anti- or pro-inflammatory characteristics. It is the combination of low diversity in bacteria, fewer butyrate-producing species, and the microbial load that renders this enterotype dysbiotic. This further proves that the metabolic network of the microbiome is highly interconnected. The dynamic properties of the microbiome make research more complicated, with confounding factors like diet, antibiotics, other medication, and the severity of the disease affecting the outcomes of such studies. Taking note of these variables is crucial to ensure a complete understanding of the relations between the microbiome and IBD.

Is it then relevant to study these enterotypes in depth? There has been some criticism of the concept of enterotypes because of its oversimplification of the microbiome. This criticism also relates to the instability of enterotypes, with environmental factors like age and diet strongly influencing the microbiome composition. Additionally, the clusters tend to form a gradient, rendering the distinction between subtypes more complex.

However, the instability of enterotypes is not necessarily a negative aspect. In the context of IBD and the Bact2 enterotype, this can be utilised to move away from microbial dysbiosis by applying a different type of diet. There are many benefits of consuming a healthier diet, especially because whole-grain carbohydrates, low-fat foods, and dairy are relatively accessible and generally beneficial foods. However, the examined dietary studies utilised either in vitro models, mice, or clinical trials with a focus on obesity (see Table 2). In the context of IBD, it is crucial for clinical trials to target UC or CD patients. Additionally, there are still issues in utilising donkey milk specifically as a treatment. Due to the low production yield, the lack of knowledge about the bioactive compounds, and the large variation in milk composition among donkey breeds, it is difficult to standardise [82]. Moreover, these findings are yet to be reproduced in human clinical trials, so the diet’s effectiveness in disease-affected patients should be investigated further.

Concerning the enterotypes, it is also important to note that, even with the clustering complexity, there seems to be a clear pattern, where one side of the spectrum is more sensitive to inflammation and the other presents a healthier profile. Adding to this, one of the most relevant findings concerning the role of enterotypes in IBD is that it can influence the effectiveness of medication. The reason for this is still unclear. Due to dysbiosis playing a crucial role, FMT could be beneficial for patients where therapeutic drugs are deemed unsuccessful. This is reflected in Lu et al. (2025), showing FMT to be more effective in IBD patients experiencing bile acid malabsorption [69]. The malabsorption of bile acids was also observed in Bact2 patients in the study by Poppe et al. (2024), again highlighting how dysbiosis affects IBD [33].

Nevertheless, to truly understand the ineffectiveness of these treatments, proper research should be conducted into the different enterotypes in the context of IBD. One of the major limitations of these studies is the lack of standardisation when clustering enterotypes. Poppe et al. (2024) used a generative model, known as Dirichlet Multinomial Mixtures (DMM), with the Flemish Gut Flora Project as the database for the stratification of the enterotype clusters [33]. This database, however, is not publicly accessible, meaning that the clustering of patients may vary depending on the study location. Moreover, not every study describing enterotypes utilises this algorithm. This can result in inconsistencies due to unaccounted for and unknown variables. When critically examining the papers discussed throughout this narrative review, the differences in cohort size, sequencing techniques, and methods of clustering vary, meaning that the definition of Bact2 is not fixed. Furthermore, most of the clinical studies reporting metabolite concentrations only looked at faecal samples, rather than mucosal samples. As faecal samples are only representative of the conditions of the gut close to the rectum, they fail to capture the conditions in other parts of the intestine. For this reason, mucosal samples present a broader image of how the microbiome truly acts during inflammation, especially for patients with CD, where inflammation can occur outside the colon. The downside of mucosal biopsies is that it is more invasive than taking a faecal sample. Ultimately, the goal of understanding the effects of enterotypes and dysbiosis in IBD is to predict how patients will respond to treatment, as well as to consider the utilisation of biomarkers. This comes with its own limitations, as enterotypes are unstable, as discussed previously. Moreover, if faecal samples are not reliable for clustering, mucosal biopsies as a diagnostic tool would be more impractical.

## 12. Further Perspectives and Conclusions

To truly understand whether Bact2 has an influence on IBD pathology, the microbiome in these patients should be tested for a causal relationship. The metabolomics study in Bact2 patients by Poppe et al. (2024) only showed the metabolic profile in retrospect, after inflammation had taken place [33]. For this reason, the links proposed in this review remain speculative regarding how the metabolic profiles of Bact2 carriers could influence inflammation and the pathology of IBD. For further research, FMT could be performed on germ-free mice using IBD patients possessing various enterotypes as donors. In this manner, it may be possible to determine whether certain conditions could lead to colitis symptoms. Importantly, to understand the effects of diet and medication on Bact2, clinical trials involving IBD patients with different enterotypes undergoing several dietary interventions would be valuable. This would allow a more detailed understanding of which food groups assist in the shift away from dysbiosis.

**Figure 6:**
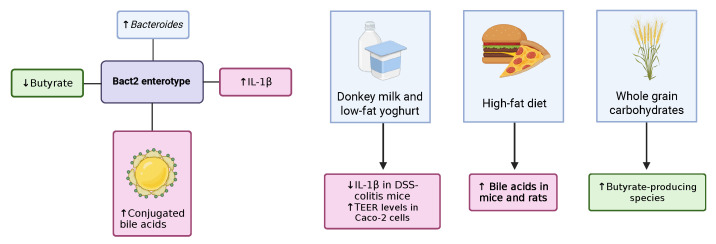
Overview of the influence of the diet on the metabolites and bacteria associated with the Bact2 enterotype. The Bact2 enterotype is characterised by an abundance of *Bacteroides* species, a decrease in butyrate, and an increase in conjugated bile acids and IL-1β. A low-fat diet can prevent high levels of bile acids, whereas dairy, such as low-fat yoghurt and donkey milk, inhibits the levels of IL-1β. Additionally, the consumption of whole-grain carbohydrates increases the levels of butyrate. Created in BioRender. Te velde, A. (2026) https://BioRender.com/you4s17.

In sum, Bact2 is an enterotype related to the malabsorption of bile acids, elevated levels of pro-inflammatory cytokines, and reduced butyrate concentrations. Together, these factors contribute to the exacerbation of inflammation in IBD. This dysbiosis can even influence the effectiveness of therapeutic treatment. However, the microbiome and its metabolism can be manipulated through diet, namely through the consumption of low-fat and plant-based foods, unrefined carbohydrates, and dairy products, such as low-fat yoghurt (see Figure 6).

## 13. Methods

The information for this study was collected using article databases such as PubMed and Google Scholar. The search terms were ‘IBD’, ‘Bact2’, ‘*Bacteroides*’, ‘Enterotypes’, ‘bile acids’, ‘butyrate’, ‘intestinal barrier’, and ‘diet’. Articles were sorted by ‘Most recent’ or ‘Best match’ and filtered to include only those written in English to ensure relevance. The reference lists of key review and research articles were also screened to identify additional relevant studies. In total, 117 articles were screened, of which a subset of 89 articles was selected for inclusion in this narrative literature review. These articles were published between May 2002 and December 2025. Randomised controlled trials, as well as meta-analyses and literature reviews, were used to investigate the mechanisms underlying Bact2 dysbiosis. Articles that lacked sufficient detail or fell beyond the scope of this paper were excluded.

## Figures and Tables

**Table 1:** Studies examining bile acid metabolism and microbial dysbiosis, comparing experimental subjects, cohort sizes, and sequencing techniques.

Paper	Experimental Subjects	Cohort Size	Main Findings	Sequencing Technique
Poppe et al. (2024) [33]	UC patients	128 individuals total (51 healthy controls, 36 UC-A and 41 UC-R patients)	Conjugated bile acids were found to be upregulated in Bact2 patients.	16S rRNA
Yang et al. (2025) [63]	DSS-induced colitis BALB/c mice	24 mice total (6 per experimental group)	Deconjugated bile acids were found to be upregulated in the plasma of diseased mice.	Illumina shotgun
Friedman et al. (2018) [64]	Healthy volunteers	24 individuals total	Administration of OCA in humans showed a decrease in bile acid production.	16S rRNA
Xu et al. (2021) [67]	Wild-type C57BL/6J mice	19 mice total (9 healthy controls, 10 treated mice)	Supplementing mice with DCA led to an increase in deconjugated bile acids.	16S rRNA
Duboc et al. (2013) [68]	IBD patients (UC and CD) during remission and flare-ups	42 IBD patients (12 CD, 30 UC, 23 active IBD patients, and 19 in remission) and 29 healthy controls	Secondary bile acids were reduced in IBD patients and more often sulphated due to a lack of sulphatase.	16S rRNA
Lu et al. (2025) [69]	IBD patients	106 IBD patients total (37 UC and 69 CD patients) and 24 healthy controls	Found an abundance of sulphated bile acids in IBD patients and BAM. FMT treatment was more successful in patients experiencing BAM.	16S rRNA

**Table 2:** Papers examining how diet can influence metabolic pathways and dysbiosis, comparing cohort sizes and sequencing techniques.

Dietary Intervention	Main Findings	Experimental Subjects	Sequencing Technique	Paper
Carbohydrate- and calorie-restricted diet, followed by a reduction in protein-enriched foods	Low microbial diversity affects how diet influences the microbiome, as it was associated with higher BMI and increased triglycerides and liver transamines.	263 healthy volunteers	Whole genome (MinION)	Alili et al. (2022) [77]
Unrefined carbohydrates	Consumption of unrefined carbohydrates leads to an increase in butyrate-producing species (Roseburia). The bran and germ contain dietary fibre, which is utilised by these bacteria and leads to a surge in their growth.	11 healthy volunteers	16S rRNA	Faits et al. (2020) [78]
Low-fat yoghurt	Low-fat yoghurt significantly reduced intestinal barrier permeability, as well as paracellular translocation and IL-8 production, after induction with IL-1β.	Caco-2 cells, induced by IL-1β	NA	Zhai et al. (2019) [80]
Donkey milk	Donkey milk shows anti-inflammatory effects on DSS-induced colitis mice via upregulation of ZO-1, occludin, MUC2. The abundance of bacteria belonging to the Firmicutes phylum also increased, which are mainly SCFA producers.	40 mice total (10 per group, with 2 control and diseased groups)	16S rRNA	Yang et al. (2025) [81]
High-fat diet (DCA)	After consumption of a Western diet, ileal and colonic inflammation and levels of bile acids increased.	19 mice total (9 healthy controls, 10 treated mice)	16S rRNA	Xu et al. (2021) [67]
High-fat diet (CA)	Administrating CA to imitate a high-fat diet caused an increase in the level of *Enterobacteriaceae* and a decrease in alpha diversity. Besides this, a decrease in bacterial density was also found in the intervention groups.	33 rats total (12 healthy controls, 9 rats in the M-CA group, 12 rats in the H-CA group)	16S rRNA	Islam et al. (2011) [83]

## Data Availability

No new data were created or analysed in this study.

## Abbreviations

The following abbreviations are used in this manuscript:

Bact2	*Bacteroides* enterotype 2
BAM	Bile acid malabsorption
BSH	Bile salt hydrolase
CA	Cholic acid
CD	Crohn’s disease
CDCA	Chenodeoxycholic acid
DCA	Deoxycholic acid
DSS	Dextrose sodium sulphate
FMT	Faecal microbiota transplant
FXR	Farnesoid X receptor
IBAT	Ileal bile acid transporter
IBD	Inflammatory bowel disease
IFX	Infliximab
IL	Interleukin
MEKK-1	Mitogen-activated protein kinase kinase kinase-1
MLCK	Myosin light-chain kinase
NETs	Neutrophil extracellular trap
NF-κB	Nuclear factor kappa-light-chain-enhancer of activated B cells
PCA	Principal component analysis
SCFA	Short-chain fatty acid
SHP	Small heterodimer partner
TCA	Taurochenodeoxycholic acid
TDCA	Taurodeoxycholic acid
TEER	Transepithelial resistance
Th17	T-helper cell 17
TLR	Toll-like receptor
Treg	Regulatory T cell
UC	Ulcerative colitis
VDZ	Vedolizumab
ZO-1	Zonula occludens 1
